# Monitoring of erosive tooth wear: what to use and when to use it

**DOI:** 10.1038/s41415-023-5623-1

**Published:** 2023-03-24

**Authors:** Saoirse O’Toole, Francisca Marro, Bas A. C. Loomans, Shamir B. Mehta

**Affiliations:** 41415202044001 Faculty of Dentistry, Oral and Craniofacial Sciences, King´s College London, Guy´s Campus, London, UK; 41415202044002 Department of Paediatric Dentistry, PAECOMEDIS Research Cluster, Gent University, Gent, Belgium; 41415202044003 Department of Dentistry, Radboud University Medical Centre, Radboud Institute for Health Sciences, Nijmegen, The Netherlands; 41415202044004 Faculty of Dentistry, Oral and Craniofacial Sciences, King´s College London, Guy´s Campus, London, UK; Department of Dentistry, Radboud University Medical Centre, Radboud Institute for Health Sciences, Nijmegen, The Netherlands; College of Medicine and Dentistry, Birmingham Campus, Ulster University, UK

## Abstract

Although we are increasingly recognising the need to assess patients for accelerated rates of tooth wear progression, it is often difficult to do so within a feasible diagnostic window. This paper aims to provide evidence-based timelines which a diagnosing clinician can expect to assess tooth wear progression in study models, clinical indices, clinical photographs and visually with intraoral scans. It also discusses new technologies emerging for the quantitative assessment of tooth wear, timelines for diagnosis, and caveats in the 3D scan registration and analysis process.

## Key points

- Reviews the aetiology of erosive tooth wear.
- Discusses new and old ways of monitoring erosive tooth wear progression.
- Highlights the timelines over which you can expect to see change using each of these methods.
- Gives an insight into the future of computer-aided diagnostics for tooth wear in dentistry.

## Introduction

The prevalence of erosive tooth wear, defined as the chemo-mechanical destruction of dental hard tissue, is increasing, particularly in the younger age cohorts.^1^ Both patients and dentists are becoming more aware of the condition. Despite this, it is often difficult to ascertain the aetiology. We snack more on acidic foods and drinks^2^ and it is often the cumulation of insults rather than one specific aetiology.^3^ Dietary acids are the most common aetiology of erosive tooth wear. A seemingly healthy diet consisting of juice with breakfast, a mid-morning apple, salad with dressing for lunch, sparkling water with lemon mid-afternoon and a fruit tea or glass of wine after dinner would represent five acid challenges per day. A single-centre case control study suggested that regular consumption of two dietary acids per day could result in tooth wear.^3^ If this daily intake becomes habitual and is combined with sleep bruxism, it is easy to see how a rapid rate of tooth wear could occur.

There has been consensus that dentists need to start screening for tooth wear more frequently and actively monitor erosive tooth wear.^4^ In addition to reducing the biological and financial cost to the patient,^5^ dentists are in an ideal position to be the signal diagnosticians for many underlying insipid health conditions associated with erosive tooth wear. We are also increasingly recognising the impact of many health conditions on erosive tooth wear, with recognised associations with gastro-oesophageal reflux disease,^6^ asthma,^7^ eating disorders,^8^ obesity,^9^ xerostomia,^10^ alcoholism^11^ and obstructive sleep apnoea.^12^ It is difficult to quantify the prevalence of silent (asymptomatic) gastro-oesophageal reflux, but population screening studies suggest that it can affect 7-10% of the population,^13^ with higher rates in those with obesity.^14^ Obstructive sleep apnoea is thought to affect 3% of women and 10% of men at 30-49 years of age, and 9% of women and 17% of men at 50-70 years of age.^15^ Diagnosed eating disorders are thought to affect 8.4% of women and 2.2% of men, although point prevalence studies for disordered eating can be as high as 19.4% for women and 13.8% for men.^16^ All of these conditions can interplay with each other, making it notoriously difficult to confidently diagnose the underlying aetiology of erosive tooth wear. As an example, gastro-oesophageal reflux disease is often responsible for high levels of intraoral acid exposure. However, it is also potentially a trigger for bruxism and the two conditions often coincide with each other. Another example is vomiting eating disorders. These can often be masked by acidic diet drink consumption which is higher in this cohort. The first line therapy for vomiting eating disorders is often selective 5-hydroxytryptamine reuptake inhibitors, which can cause both xerostomia and bruxism. If aggressive oral hygiene is also performed, it is difficult to separate one aetiology from the next unless there is active monitoring with patient feedback. Finally, for restorative treatment, understanding the aetiology and rate of tooth wear will aid in determining whether to intervene and material choice.

With recent advances in technology, the monitoring of tooth wear can now be classified into qualitative or quantitative monitoring. Qualitative monitoring occurs when patient data at baseline and at a future time point are subjectively assessed for significant change, often with a simple yes/no outcome. However, when using a scale, such as present in a clinical index, it becomes semi-quantitative. Quantitative monitoring involves the measurement of change in patient data with a numeric outcome of wear progression. This paper discusses qualitative and semi-quantitative measurement methods together and quantitative methods separately, outlining the advantages, diagnostic window and limitations of each method. Accurate assessment of early wear changes often necessitates the ability to inspect changes in depth, texture, translucency and colour. It is worth bearing in mind that advanced quantitative analysis, whereby accurate models have been scanned using profilometers capable of analysing change at sub-micron level within a university setting, have determined normal wear progression over six months to be in the region of 15 microns.^17^^,^^18^ Given that the depth resolution of the human eye is circa 200 microns,^19^ this would suggest that human assessment of depth alone is an ineffective method to diagnose wear in a feasible diagnostic window. We must therefore assess the limitation of each method with respect to this.

## Qualitative/semi-quantitative monitoring of erosive tooth wear progression

### Study models/dental stone casts

Taking study models at baseline and at future appointments is often recommended as a method to monitor tooth wear progression.^20^ However, visual inspection of changes on casts necessitates the longest diagnostic timeline. Stone casts cannot provide information on tooth colour or translucency. Early texture changes will be reliant on the accuracy of the impression and casting process.^21^ Accuracy for impression materials, independent of operator error, can range from 11-67 microns.^22^ This is akin to the level of wear that we are trying to detect over a six-month period. Wear at a 67 micron rate is a high level of progression and wear at an 11 micron rate over six months would be a physiological level of progression for this time period.^23^ When operator error is considered, wear diagnosis can be very difficult. The minimum time period which has been used in research to detect change on stone casts has been two years.^24^ However, one retrospective analysis of casts over a nine-year period observed that it required 4-5 years in order to confidently diagnose an accelerated rate of progression^25^ when operator error is taken into consideration. Finally, the exposure of dentine, an important assessment criteria for many of the clinical indices, cannot be accurately assessed on study casts.^26^^,^^27^ Given this information, study models are often more of a patient education tool rather than a method to monitor wear within a feasible diagnostic window.

### Clinical indices

The use of clinical indices has been increasing in recent years, with the increasing adoption of the Basic Erosive Wear Examination (BEWE)^4^ and the Tooth Wear Evaluation System.^28^ Clinical indices offer a higher chance of seeing change, as one can observe slight changes in texture, translucency and colour. Direct comparisons between studies are difficult, as different indices are more sensitive than others. For instance, the Smith and Knight index calculates wear at the 33% and 66% levels, while the BEWE is at 50%, and the latter does not assess dentine exposure. Despite this, clinical examinations using indices may be more sensitive when measuring wear progression over a shorter period, particularly when compared to evaluation of study casts. El Aidi was able to observe statistical differences at 18 months using indices clinically in children,^29^ whereas Johansson *et al.* was unable to detect statistical differences at 18 months using study models.^26^ Dugmore and Rock observed using clinical examinations that wear progressed in 26.8% of participants over two years,^30^ whereas Bartlett observed mild tooth wear progression on relatively few surfaces when assessing orthodontic models over the same time period.^24^

There are three studies, to the authors' knowledge, that performed quantitative assessment of erosive tooth wear progression in addition to grading using indices on casts. In the majority of cases, erosive damage was subclinical over a time period of one year to 18 months.^18^^,^^31^^,^^32^^,^^33^ Al-Omiri observed that the Smith and Knight Index was unable to monitor tooth wear over a six-month and one-year period.^32^^,^^33^ Chadwick *et al.* did not observe visual differences after 18 months using a Ryge index^31^ and Rodriguez *et al.* did not observe statistical clinical difference on study casts using indices over a one-year period.^18^ Therefore, 18 months is probably the diagnostic interval for assessing change using clinical indices.

Effective use of clinical indices relies upon the ability to consistently score similar levels of wear internally (intra-operator reliability) and with others (inter-operator reliability).^34^ One study performed in NHS practice demonstrated that the inter-operator agreement on the BEWE was moderate, which can limit its use for diagnostic and monitoring purposes.^35^ However, simple online training can improve this significantly.^36^ As a practice screening tool, identifying and recording wear with a simple clinical index, such as the BEWE, is a quick and cost-effective way to document wear.

### Clinical photographs

Authors have argued that clinical photographs have the same level of accuracy at detecting tooth wear as study models,^27^^,^^37^^,^^38^ although no longitudinal studies utilising this method have been done to date. Clinical photographs also represent a fair option, offering levels of accuracy and diagnostic timelines similar to that of clinical indices, with the advantage that you can discuss and compare them with the patient. Research has shown them to offer higher diagnostic quality than study models.^38^ It is more difficult to gauge depth and texture on clinical photographs compared to a clinical exam and accuracy will often depend on the skill of the clinician to take good photographs at a consistent angle. Although it remains untested to date, a diagnostic window of 18 months may be feasible with clinical photographs, depending on the skill and consistency of the photography.

### Intraoral scanners

Intraoral scans allow assessment of depth, texture, and to a lesser degree, colour and translucency. Marro *et al.* scanned the study casts of adolescents taken at baseline and two years later. The group observed that visual inspection of scans of the casts to be more sensitive at diagnosing wear progression than visual inspection of the casts alone.^39^ Our ability to zoom in on areas of interest and gauge change from multiple dimensions is of benefit. The minimum time period that wear progression has been assessed on intraoral scans to date is two years;^39^ however, this is probably due to the novelty, rather than the capability of diagnostic potential, and the ability to visually assess changes is likely to be closer to that of clinical indices at 18 months. The true benefit of intraoral scans lies in our ability to use them for the quantitative measurement of wear progression. Using the same dataset but analysing them quantitatively, Marro was able to establish cut off points for high rates of wear progression.^40^ The quantitative monitoring of tooth wear will be discussed in the next section.

## Quantitative monitoring of tooth wear

Until now, quantitative assessment of tooth wear has been limited to a university setting. This has involved taking accurate study models, scanning them with a profilometer and measuring the change using metrology comparison software. This has facilitated the measurement of quantitative wear over six months^41^ and one year.^17^^,^^18^^,^^31^ The average wear value of 15 microns over a six-month period is at the detection threshold of advanced laboratory equipment^42^ and a systematic review recommended 25 microns as a more realistic detection threshold.^42^

There are two areas limiting our ability to measure tooth wear quantitatively in primary care. The first is the resolution detection of the primary care scanners. However, the second, and greatest source of inaccuracy, is the registration and measurement algorithm for combining the two scans taken at separate time points and analysing them. From a measurement point of view, there are no fixed intraoral reference points for comparison, which means that we need to rely on registration programmes to align scans and measure changes. Although this technology has potential to revolutionise the primary care monitoring of wear, there are still caveats associated with this form of monitoring. 

We often do not know the exact mechanism of registration and analysis in the software, which will cause measurement errors, potentially to the same agree as the biological change. For example, most registration algorithms work by minimising the distance between similar scan areas. However, when there is an area of large change, for example, substantial wear, no algorithms to date can recognise this and the software will minimise the wear by bringing the two scans into the closest possible proximation. This will often result in areas of positive data or tissue gain, as the whole scan is tilted to minimise changes. The result is an inaccurate registration with underestimation of the wear.^43^

Unfortunately, the greater the wear, the more inaccurate a best fit scan registration will be. You can help to mitigate this inaccuracy by registering the surface on selected areas which are deemed to not have undergone substantial change^43^ and this methodology can increase accurate *in vivo* measurements.^44^ However, this requires estimation of the wear areas, additional operator input and analysis time. At the time of print, there are dental commercial software that offer semi-quantitative assessment of wear, such as TRIOS Patient Monitoring (3Shape, Denmark) and OraCheck (Dentsply Sirona, UK). Although several features are available, such as quantitative determination of height and volume, it is relatively easy for software to mask errors by only showing the negative data. Be sceptical of any data presented by software that do not present a scale bar demonstrating positive (tooth surface gain data) and negative (tooth surface loss data). The level of 'gain' data present is often an indicator of how accurate the registration is as teeth cannot gain tissue. However, due to the flaws in registration, it is currently difficult to accurately determine changes less than 50 microns. This may represent a larger wear rate than can be detectable with advanced laboratory equipment but is a useful and accessible clinical tool.

**Fig. 1:**
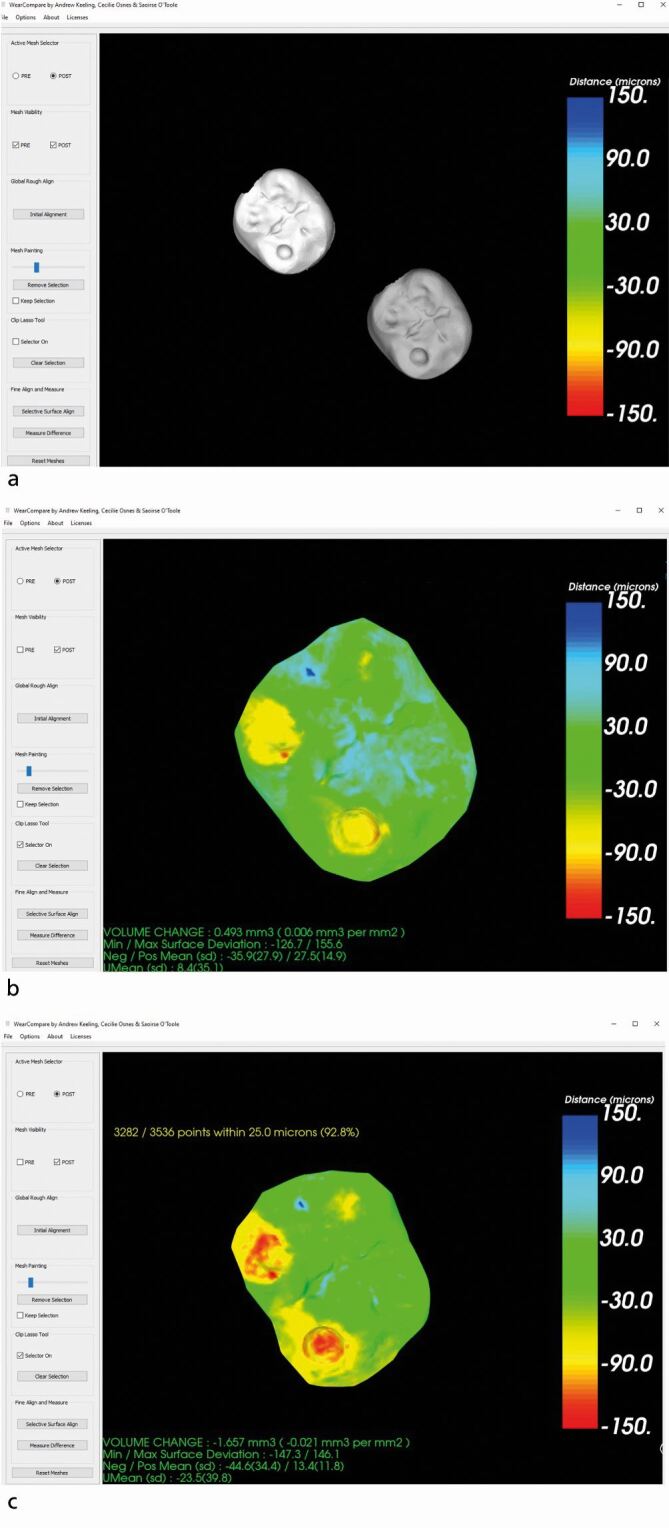
Wear analysis on a lower left second molar using WearCompare. The top image shows two intraoral scans taken three years apart. Deepening of the concavities can be observed by the naked eye. The middle image shows wear analysis of the tooth after a simple registration. Wear facets can be seen in yellow but areas of tissue gain (blue) remain on the surface. This is a flawed registration. The bottom image shows wear analysis after a selective surface registration, aligning only on the lingual and buccal aspects of the tooth. Deepening and widening of the concavities can be seen (red and yellow areas) in addition to wear on the buccal occlusal surface. The areas in blue (tissue gain) are less prevalent on the surface. This demonstrates the importance of an accurate scan and accurate registration

A purpose-built freeware, WearCompare, is also available for use with intraoral scans. Designed in a collaboration between King's College London and Leeds Dental University,^45^ it is a simple application which requires selecting the registration surfaces separately to the areas for analysis. However, the process is most accurate when done tooth by tooth, is time consuming and operator dependent. An illustration of the registration and measurement process is shown in Figure 1. There is a need for improved algorithms, ideally biologically informed, with machine learning on big data, to improve our ability to accurately register scans to detect wear and provide computer-aided diagnostics. This is the focus of several research groups and it is only a matter of time before we can offer this service to our patients. An overview of all diagnostic intervals for each method of monitoring discussed is shown in Table 1.

**Table 1:** When you are likely to see changes when monitoring erosive tooth wear

Method of monitoring	Diagnostic interval
Study casts	3-5 years
Clinical indices	18 months
Clinical photographs	18 months
Visual changes on intraoral scans	18 months to 2 years
Wear analysis on intraoral scans	6 months to 1 year

## Conclusion

For reasons outlined above, little is known about the rate of normal and pathological progression rates of tooth wear. Some will have gradual change over their lives with little impact on the health of the dentition. Others will undergo rapid change with compromises to aesthetics and tooth longevity. For the latter group, there is a duty of care to identify tooth wear at an early stage in addition to safeguarding against litigation. By simply taking a clinical index, a photograph, or an intraoral scan to document wear, we can potentially diagnose accelerated tooth wear, an underlying health condition and protect ourselves from litigation.

Active screening and monitoring tooth wear should play a part in every new patient clinical examination. Diagnostic windows for detecting qualitative change on study models, clinical indices, clinical photographs and intraoral scans range from 18 months to two years. It is likely that commercial tooth wear analysis software will override the need to record a clinical wear index if you take intraoral scans. However, until then, or if working with an analogue workflow, documenting a clinical index is a prudent measure. Clinical photographs remain part of the diagnostic process and are less likely to be replaced in the future. Currently, different software exist for the quantitative measurement of tooth wear, with intraoral scans with potential to diagnose active wear in six months. However, current problems with scan registration accuracy and measurement limit their diagnostic potential. Given this is likely to improve, intraoral scans can be recommended to patients as an effective way to monitor wear progression; qualitatively for now, and quantitatively in the near future.
